# Gaining the Upper Hand on Pulmonary Drug Delivery

**DOI:** 10.4172/2329-6887.1000118

**Published:** 2014-03-01

**Authors:** Jean Tyrrell, Robert Tarran

**Affiliations:** 1 Cystic Fibrosis/Pulmonary Research and Treatment Center, North Carolina, USA; 2 Department of Cell Biology and Physiology, University of North Carolina, North Carolina, USA

## Abstract

Asthma, Chronic Obstructive Pulmonary Disease (COPD) and Cystic Fibrosis (CF) are all pulmonary diseases which are characterized by chronic inflammation and an increase in mucus production. Excess mucus in the airways correlates with pathophysiology such as a decline in lung function and prolonged bacterial infections. New drugs to treat these chronic respiratory diseases are currently being developed and include both inhaled and orally administered compounds. Whilst oral drugs may be easier to administer, they are more prone to side-effects due to higher bioavailability. Inhaled compounds may show reduced bioavailability, but face their own unique challenges. For example, thick mucus in the respiratory tracts of asthma, CF and COPD patients can act as a physical barrier that impedes drug delivery. Mucus also contains a high number of enzymes and proteases that may degrade compounds before they reach their site of action. Furthermore, some classes of drugs are rapidly absorbed across the respiratory epithelia into systemic circulation, which may limit their duration of action and/or cause off-target effects. This review discusses some of the different treatment options that are currently available and the considerations that need to be taken into account to produce new therapies for the treatment of chronic respiratory diseases.

## Introduction

Asthma, chronic obstructive pulmonary disease (COPD) and cystic fibrosis (CF) are all pulmonary diseases that are characterized by chronic inflammation and an increase in mucus production [1–3]. In CF, mutations in the CF transmembrane conductance regulator (*CFTR*) gene result in abnormal chloride secretion. This leads to dehydrated mucus and a failure of mucus clearance, making the airways prone to bacterial infections, chronic inflammation and chronic neutrophilia which result in pulmonary destruction and morbidity [4,5]. COPD and asthma have different underlying pathophysiology, with COPD representing an exaggerated inflammatory response to a chronic inhaled toxicant, usually tobacco smoke, and with asthma representing a genetic disposition to abnormal inflammatory responses to environmental stimuli such as dust mite allergens [6]. However, both the chronic bronchitis forms of COPD and asthma both present with mucus obstruction of the airways which can be fatal [7–10]. Excess mucus in the airways correlates well with disease pathophysiology such as a decline in lung function and prolonged bacterial infections [11–13]. At present there is no cure for any of these diseases. However, a number of treatment options such as inhaled antibiotics and mucolytic drugs are available to ease some of the respiratory symptoms. For the treatment of CF, oral CFTR correctors/potentiators that seek to pharmacologically correct common disease-causing CFTR mutations are being developed [14,15]. Asthma is typically managed with inhaled corticosteroids and β2 agonists [16]. Numerous potential therapies are also currently under development for COPD. In this mini-review, we discuss the delivery routes that are available for dosing the lungs, and the challenges encountered.

## Systemic vs. Inhaled Delivery

Drugs to treat chronic respiratory diseases such as asthma, CF and chronic bronchitis have been available for many years and include both inhaled and orally administered compounds. When treating pulmonary diseases, there are several advantages of using inhaled drug delivery over systemic drug delivery; with inhalation, there is a rapid clinical response, systemic side effects are often minimized and inhaled drugs bypass barriers to therapeutic efficacy such as poor gastrointestinal absorption and first-pass metabolism in the liver [17]. Examples include inhaled antibiotics, corticosteroids, β2 agonists and mucolytics. Inhaled corticosteroids and beta agonists are the primary treatment for mild to moderate asthma as they can control asthma symptoms, improve lung function, and decrease the risk for exacerbations immediately as they reach the lungs quickly [18,19]. A downside to inhalation therapy is that the amount of drug delivered is limited by the efficiency of the device. Nebulizers deliver more drug than hand held inhalers but are time-consuming to the point that patient adherence can be risked. As a case in point, CF patients spend more than 1h/day using a nebulizer, which has been suggested, is the limit of patient compliance [20,21].

In contrast, systemic drug delivery can be slower, there may be more off-target effects since the drug has better access to other organs, and a greater chance of the drug being metabolized/excreted before it can work on the lungs. Oral delivery is useful for patients with severe disease and the elderly who, due to technical difficulties, cannot properly use inhalers or nebulizers reliably and therefore may not receive the effective dose. Also, drugs with slow pharmacokinetics allow for sustained and higher drug concentrations in the lung. For example, intravenous antibiotics are used to treat severe lung infections [22]. Oral steroids such as prednisone are often prescribed for severe asthma attacks and serve to dampen down lung inflammation. However, their systemic delivery can have numerous side effects including glaucoma, fluid retention, increased blood pressure and mood swings [23–25].

Roflumilast is a type 4 phosphodiesterase inhibitor that is administered orally for the treatment of severe COPD. It is an anti-inflammatory agent designed to target both the systemic and pulmonary inflammation associated with COPD and has been shown to improve lung function [26]. Since roflumilast is introduced orally, even though the site of action are the pulmonary epithelia, which are accessible by inhalation, it has been reported to cause mild to moderate diarrhea in 10% of the population, presumably due to elevated cAMP levels in the gastrointestinal tract [27–29]. Decreased weight, psychiatric disorders, nausea, headache, dizziness and decreased appetite have all been reported during clinical trials in 0.4–5.2% of the population [29]. However, absorption of roflumilast after oral administration is rapid and after a single dose the bioavailability is ~80% [30]. Roflumilast is not affected by food and metabolism occurs via the liver (54). Since COPD predominantly affects the elderly, oral roflumilast may be the preferred route for severe COPD sufferers and may afford for a sustained therapeutically useful dose of roflumilast in the lungs.

## Mucus as a Barrier to Inhaled Drugs

Airway surface liquid lines normal airways and acts as a lubricant for efficient mucus clearance [4,31]. The mucus component is normally thin (typically ~7 microns), ~2% solids and easily cleared by ciliary beating or cough clearance. In CF and chronic bronchitis lungs, the mucus layer is dehydrated due to abnormal ion transport (values as high as ~20% and ~8% solids have been reported in CF and chronic bronchitis respectively [32,33]. In CF, the mucus layer also contains high levels of DNA and actin polymers, the debris of an aggressive neutrophilic inflammatory response to infection [34,35]. Thus, while thickened mucus attempts to protect the body by trapping inhaled pathogens/particles, it also becomes a limiting factor for inhaled aerosol-based therapies and can act as a physical barrier that impedes drug delivery. Characteristics of aerosols that affect their ability to penetrate mucus include size, particle charge and solubility. The mucus barrier can limit the permeability of hydrophobic drugs [36]. Dawson et al. [37] have shown that neutrally charged polystyrene particles less than 200 nm in diameter can travel faster than charged particles through CF sputum. Indeed, in our own studies of airway surface liquid metabolism, we sometimes use fluorescent microspheres to label the mucus layer, and have found that negatively charged 100 nm beads “stick” to the mucus allowing us to image and track it [38]. Suk et al. [39], have estimated that the pore size of CF sputum is ~140 ± 50 nm (range: 60–300 nm). Thus, drug particles up to 200 nm can travel through this mucus when coated with low molecular weight polyethylene glycol (PEG) [39]. In another study, transport of these nanoparticles was examined in CF sputum that was treated with *N*-acetylcysteine, which increased the average spacing within the sputum from ~140 ± 50 nm to ~230 ± 50 nm [40]. These studies show that promising advances being made towards overcoming the hurdles to mucopenetration during pulmonary drug delivery. However, when formulating a drug against diseases of mucus dehydration for inhalation, particle size and charge remain major factors to consider.

## Metabolism of Drugs in the Lung Lumen

During a proteomic analysis of airway surface liquid, Candiano et al. [41] found that out of 175 proteins detected, enzymes and immune-related proteins accounted for ~65 % of all proteins. Thus, before any therapeutic effect can even take place, drugs may also be metabolized by ectoenzymes found either in the plasma membrane of pulmonary epithelia or in airway secretions. Ectoenzymes can either be transmembrane proteins, membrane anchored or secreted but all are active in the lung lumen. Examples of ectoenzymes include serine and acid proteases (e.g. prostasin and cathepsin B) and nucleotidases such as the 5′-nucleotidase (CD73) [42]. Given the high amount of enzymes present, it can be suggested that a number of these may metabolize respiratory drugs before they can have their desired effect, with peptide- and nucleotide-based drugs being especially vulnerable to metabolism. Furthermore, additional proteases are released into the lung during chronic inflammation/infection (e.g. neutrophil elastase), suggesting that drug stability must be assayed under conditions that closely mimic the diseased state. Bacterial pathogens such as *P. aeruginosa*, one of the predominant pathogens involved in CF lung infections, release additional proteases such as alkaline protease contributing to the increase in lung lumen proteases in CF airways [43].

Secretory leukocyte peptidase inhibitor (SLPI) is an endogenous, secreted serine protease inhibitor. It protects the lungs from excessive tissue damage caused by leukocyte proteases released during inflammation and may also possess anti-inflammatory effects. However, its clinical action has been limited due to enzymatic cleavage by cathepsins within the lung [44]. Gibbons et al. [45] demonstrated that liposome encapsulation of rSLPI can improve stability and potentially reduce the level and frequency of dosing required. Thus, when designing new inhaled therapies enzymatic degradation in the lung lumen should be taken into account.

## Rapid Absorption of Xenobiotics across Lung

Although inhaled drugs can exert beneficial pharmacological effects on the lungs and have been used effectively for numerous years, in some cases systemic absorption may occur following inhalation, which can have unwanted side effects. Drug absorption across the lung into the bloodstream is regulated by a thin alveolar–vascular permeable barrier [17]. The ideal inhaled drug should be retained in the lung whilst the desired pharmacological action occurs, without being absorbed into systemic circulation. Due to the unique nature of the lungs, the alveolar surface is very thin and the lungs are highly vascularized. Thus, the lungs provide a large surface area for absorption of drugs into systemic circulation. Some lipophilic compounds may directly diffuse down their concentration gradient into the circulation. For example, the lung is a highly efficient route for passive nicotine absorption [46]. Interestingly, the lungs have evolved several mechanisms to remove xenobiotics including organic anion transporters (e.g. OAT1, SLC22A6) [47] organic cation transporters (e.g. OCT1; SLC22A4) [48] and p-glycoprotein (MDR1) [49]. As such, xenobiotics that are recognized by these transporters are taken up into systemic circulation so that they can be metabolized by the liver and excreted via the kidneys.

In the absence of CFTR, Na^+^ absorption through the epithelial Na^+^ channel (ENaC) is enhanced in CF airways, which further contributes to mucus dehydration by depleting the airways of salt and water [50]. ENaC antagonism with inhaled amiloride was proposed as a remedy for mucus dehydration in CF airways. Amiloride blocks ENaC with sub-micromolar potency, but was originally designed as an orally-delivered diuretic. However, amiloride failed to have any impact on CF lung disease [51,52]. The reason for this is that amiloride has an extremely short half-life in the airways after inhalation. In vitro, when deposited on airway surfaces, the half-life was ~9 min [53], since amiloride is rapidly absorbed by organic cation transporters [54]. Given that amiloride is a diuretic, there is concern over its use as its rapid absorption through the respiratory tract may cause an effect on systemic sodium and potassium balance via actions on the kidney [55]. Indeed, intranasal installation of amiloride to mice caused them to lose ~10% of their body weight/day due to urine excretion [56].

Salmeterol is a β2 agonist that is used for the treatment of asthma and COPD that is poorly absorbed by the lung. Despite exhibiting robust pharmacological effects, including relaxation of smooth muscle, plasma concentrations of salmeterol are extremely low or even undetectable after inhalation. In contrast, systemic salmeterol would lead to changes in heart rate, QTc interval, and plasma potassium and glucose concentrations [57,58]. Long-acting β2 agonists are frequently taken with inhaled corticosteroids. However, Horvath et al. have recently shown that corticosteroids can affect expression levels of the organic cation transporters that are responsible for absorbing β2 agonists, thus dramatically affecting their bio-distribution with time [59].

Thus, direct drug delivery into the lung has proven to be advantageous for the treatment of chronic lung diseases due to the lower risk of side effects. However, such drugs must be poorly absorbed across the epithelia. More recently, groups are taking advantage of rapid pulmonary drug absorption and are investigating how to treat systemic diseases through pulmonary drug delivery since this allows for drug delivery that bypasses the stomach [60].

## Conclusions

When treating pulmonary diseases such as asthma, COPD and CF, inhalation may be the best route of administration due to the rapid clinical response and the high doses of drug that can be administered to the disease site with limited off-target effects. However, a number of considerations need to be taken into account when designing new inhaled therapies. Particle size, particle charge and solubility and physical characteristics of particles need to be considered as well as the ability of these particles to withstand the defense barriers presented by the lung itself. An ideal drug should also have a long half-life and minimal to no absorption across the pulmonary epithelium. Inhaled compounds must also be able to withstand degradation by ectoenzymes within the lung. A final consideration is their ability to penetrate the thick mucus often associated with respiratory diseases. Inhaled therapies may need to be administered alongside mucolytic drugs to aid penetration through mucus. Getting all of these factors to successfully align can lead to successful drug deposition within the lung followed by drug elimination without any systemic side effects.
